# Some habits are more work than others: Deliberate self‐regulation strategy use increases with behavioral complexity, even for established habits

**DOI:** 10.1111/jopy.12926

**Published:** 2024-03-07

**Authors:** Blair Saunders, Kimberly R. More

**Affiliations:** ^1^ Division of Psychology University of Dundee Dundee UK; ^2^ Department for Health University of Bath Bath UK

**Keywords:** behavior change, goals, habit, health, self‐regulation

## Abstract

**Objective:**

We tested the hypothesis that complex behaviors are commonly supported by self‐regulation strategies, even when those behaviors are supported by strong instigation habits.

**Background:**

Goal‐directed and habit‐mediated processes arise from separable systems that have been suggested to seldomly interact.

**Results:**

Self‐regulation strategy use was lower for habitually instigated simple behaviors compared to nonhabitually instigated simple behaviors. However, participants' use of self‐regulation strategies increased with the increasing complexity of behaviors, even when complex behaviors were habitually instigated. The difference in the extent of strategy use between habitually and nonhabitually instigated actions was absent when behavioral complexity was particularly high.

**Conclusion:**

These results point to a qualitative distinction—while simple behaviors may progress in a relatively automatic and unthinking manner, complex behaviors receive frequent support from self‐regulation strategies, even if they are instigated habitually.

## 1 INTRODUCTION

Regular engagement in beneficial behaviors drives long‐term health and wellbeing. You can develop the habit of eating “an apple a day” by simply consuming the fruit every day in the same place or time. Other health behaviors are less straightforward. You might habitually fill half of your dinner plate with vegetables, cycle to work, or recycle, yet the execution of such complex acts is typically more effortful and involves multiple steps (Phillips & Mullan, 2022). We investigated whether such variation in behavioral complexity is related to differences in the psychological processes that support behavioral engagement. Here, complex behaviors are differentiated from simple behaviors in that they are more effortful, involve distinguishable procedural steps, and require both more time to enact and potentially more planning to execute (Boynton, 2005; Mullan & Novoradovskaya, 2018; Phillips & Mullan, 2022). We predicted that habitually selected simple behaviors would be highly automatic and executed without much goal‐directed support, while more complex behaviors would draw upon more support from self‐regulatory processes, even if such behaviors were selected habitually.

There is a dividing line in psychological science between goal‐directed processes that are typically intentional, capacity‐limited, and slow; and habitual processes that are automatic, fast acting, and triggered by context rather than goals (Hofmann et al., 2009; Kahneman, 2011; Metcalfe & Mischel, 1999). Self‐regulation refers to a set of goal‐directed processes that direct thoughts, feelings, and behaviors toward outcomes that are instrumental to achieving an intended state while simultaneously diverting from conflicting actions (Carver & Scheier, 1998; Inzlicht et al., 2021). Numerous tactics can be used to facilitate goal progress (Duckworth et al., 2016, 2019; Hennecke & Bürgler, 2020). If you intend to get fit, then you might set a measurable goal (e.g., to run 5K in 30 min), monitor your progress (e.g., use a run‐tracking app), seek social support (e.g., join a running club), and motivate yourself with appraisals (e.g., “you'll feel great when you are in better shape”). This nonexhaustive list of self‐regulation strategies defines tactics that act on one's mental, social, and physical environment to encourage thoughts, feelings, and behaviors that facilitate progress toward personal goals (Fujita et al., 2020; Hennecke et al., 2019; Lopez et al., 2021; Milyavskaya et al., 2021; Williamson & Wilkowski, 2020).

One pitfall of self‐regulation is that it is fallible. Individuals are often unwilling or unable to engage in cognitive effort (Kool & Botvinick, 2014) to enact an additional process (e.g., cognitive reappraisals) that steer themselves toward their goals. Overreliance on self‐regulation can also leave a person susceptible to temptation when their cognitive capacity is otherwise occupied (Westling et al., 2006). In contrast, once established, habitual behaviors can be instigated and/or executed automatically (Wood & Neal, 2007), with instigation habits (i.e., automatically deciding to do) being more strongly related to the frequency of behavioral engagement than execution habits (i.e., automatically doing; Phillips & Gardner, 2016). Habits develop when behaviors are repeatedly rewarded after a specific cue (Phillips et al., 2016), meaning that well‐established habits can trigger behaviors stored in procedural memory without mediation by goal‐directed processes (Wood & Neal, 2007; Wood & Rünger, 2016).

While the time taken to form a habit depends on behavioral complexity (Buyalskaya et al., 2023; Lally et al., 2010), intervention research has found that habits can form within a relatively short period of time, even for complex behaviors. For example, Phillips et al. (2019) found that nutrition habits can form within 4‐weeks when participants were given an action and coping planning intervention. Similarly, with intervention, physical activity habits can form within 1 to 8 weeks (Hamilton et al., 2019; Kaushal et al., 2017). Further observational research has demonstrated that new gym goers form an exercise habit with behavioral repetition occurring over 6 weeks (Kaushal & Rhodes, 2015). Though the aforementioned studies do not distinguish between instigation and execution habits, they highlight that habits can be formed prior to individuals reaching the maintenance phase of behavior change where relapse becomes less likely (Prochaska & DiClemente, 1982). Establishing habits, especially instigation habits (Phillips & Gardner, 2016), for healthy behaviors is a boon to the individual as automaticity makes behavior less vulnerable to fluctuations in motivation, fatigue, and competition for cognitive capacity compared to relying on self‐regulation (Bargh, 1994; Lally & Gardner, 2013).

Although goal‐directed behavior and habits could be cast in mutually antagonistic roles, they can also be understood as separable systems that interact in limited circumstances (Gardner et al., 2020; Wood et al., 2022). Self‐regulation initiates behaviors before habits are established (Monge‐Rojas et al., 2021; Sniehotta et al., 2005), steers behaviors when habitual cues are absent (Gardner, et al., 2020; Triandis, 1977), and can override undesirable habits that conflict with other goals (Charlesworth et al., 2022; Gardner, 2015). Further, self‐regulation can be routinely practiced to such an extent that engaging in self‐regulation itself might become habitual (e.g., habitually reminding yourself of the positive consequences of exercise; Gillebaart & de Ridder, 2015). Such “effortless self‐regulation” is distinct from the habitual instigation of behavior itself as self‐regulation still requires an indirect, secondary processing step (e.g., “reappraisal”) to encourage the behavior (e.g., “run”) that is consistent with a personal goal (e.g., “be fit”), rather than the behavior itself being triggered by the context. Thus, the need for self‐regulation is suggested to recede dramatically once habits become established (Triandis, 1977; Zhang et al., 2022).

### 1.1 The current study

The current research investigated if habitual instigation of behavior is always associated with greatly diminished self‐regulation. Habitual instigation was chosen as, unlike execution, it predicts the frequency of both simple and complex behaviors (Gardner, 2022) and does not undermine the variety needed for the optimization of several complex behaviors, such as having a varied diet or engaging in both cardiovascular and strength‐based physical activity (Phillips et al., 2019). Many health promoting behaviors are complex in that their execution involves separable steps, and they require time and potentially planning (Boynton, 2005; Mullan & Novoradovskaya, 2018; Phillips & Mullan, 2022). Complex behaviors can be contrasted with simple behaviors that take fewer steps and time to enact and have a relatively one‐to‐one mapping between a specific action and the behavior (e.g., taking a vitamin). Thus, while simple behaviors may be instigated and executed automatically, complex behaviors are unlikely to be exclusively habitual (Hagger, 2020). Yet, the composition of complex behaviors does not preclude them becoming habitual (e.g., Phillips et al., 2019). For example, cues may trigger the habitual instigation of complex behaviors (Phillips & Mullan, 2022), and the execution of complex behaviors can become habitual once highly practiced (Jenkins et al., 1994). Whether maintenance of complex behaviors is more likely to be supported through other mechanisms, such as self‐regulation strategies in addition to instigation habits, however, is a question that remains unanswered (Phillips & Mullan, 2022). The nature of complex behaviors might make them more likely to generate conflicts, for example, when selecting among substitutable means (e.g., running or cycling), or if the effort required to engage complex behaviors conflicts with rewarding alternatives (e.g., exercising vs. watching TV). Multi‐step complex behaviors that are habitually instigated are also vulnerable to a single step going wrong (e.g., misplaced running shoes) and changes in circumstances (e.g., an unexpected work deadline or family obligation; rain impeding an outdoor run; Gardner, 2015). Therefore, overcoming such conflicts or setbacks may require deliberate self‐regulation to override temptation, regulate emotions, or motivate the individual to a greater extent for complex than simple behaviors (Botvinick et al., 2001; Hennecke et al., 2019; Wood & Neal, 2007).

Although individuals may report strong instigation habits for complex behaviors, we hypothesized that these behaviors would be supported by self‐regulation strategies to a larger extent than equally habitually instigated simple behaviors. If complex behaviors are indeed realized through frequent collaboration of habitual processes with nonhabitual processes, this would point to a high degree of complementarity between fast and slow processes for behaviors often targeted by public policy and behavior change interventions. Healthy eating, exercise, and sustainable travel are all complex behaviors related to global concerns to reduce ill health (Scarborough et al., 2011) and tackle climate change (e.g., Brand et al., 2021).

We used a repeated‐measures design whereby participants identified personally relevant simple and complex behaviors that were either relatively new to them (i.e., the initiation phase of behavior change; Prochaska & DiClemente, 1982) or already established (i.e., the maintenance phase of behavior change; Prochaska & DiClemente, 1982). We predicted that established behaviors would be associated with higher instigation habit strength than nonestablished behaviors (hypothesis 1), and that complex behaviors would be associated with an increased use of self‐regulation strategies than simple behaviors (hypothesis 2). Central to the current work, it was further predicted that although self‐regulation strategies would be negatively associated with instigation habit strength, their use would still be nonzero in supporting simple instigation habits (hypothesis 3a), and self‐regulatory support would become increasingly frequent for instigation habits for complex than simple behaviors (hypothesis 3b).^1^

## 2 METHOD

### 2.1 Participants and procedure

An a priori power analysis indicated that 239 participants would be needed to achieve 90% power (*α* = 0.05), using a small effect size (Cohen's *d*_z_ = 0.1). 239 participants with complete data were recruited through Prolific (www.prolific.co, Prolific, n.d.). Prolific users were eligible to participate if they resided in the United Kingdom and were at least 18 years old. Our survey was constructed and hosted on Gorilla (www.gorilla.sc; Anwyl‐Irvine et al., 2019). The study consisted of a baseline intake survey (~30 min), and two weekly surveys – administered at seven and 14‐days post baseline (~10 min each). Each weekly survey needed to be completed within 48 h and was sent at 9 am British Summer Time with a reminder sent 24 hrs in advance. Participants were compensated £7.50 per hour, totaling £6.25 if all surveys were completed. Incomplete datasets were not retained or analyzed as they were not compensated through Prolific. Data collection lasted for 50 days (May 9, 2022 to June 28, 2022).

238 participants had complete data and passed at least 50% of the four random response checks that were presented in the baseline survey. One participant had missing data only for self‐reported automaticity and behavioral complexity at the second follow‐up for the complex nonestablished behaviors. This participant was excluded from all analyses that aggregated across follow‐ups (*t*‐tests) but not for nonaggregated analyses (multilevel models). This procedure was reviewed and approved by the University of Dundee ethics committee and all participants provided informed consent. On average, participants were 41.38 years of age (*SD* = 12.56), 63% identified as female and 37% as male.

### 2.2 Measures

**FIGURE 1:**
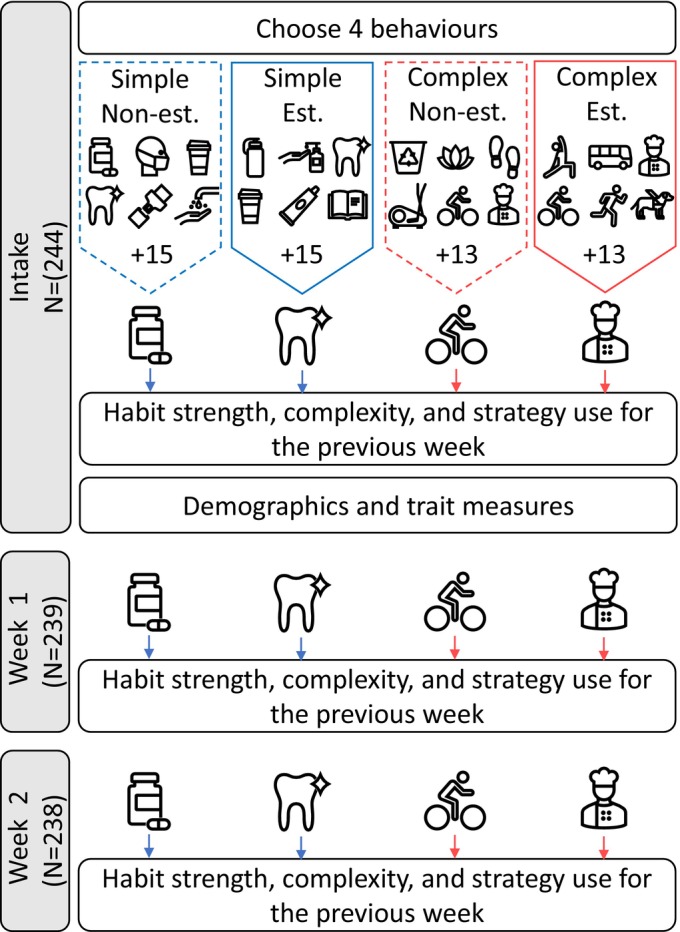
Est. = Established; Non‐est. = nonestablished. +13 and + 15 indicate the number of additional behaviors available as options.

See Figure 1 for an overview of the study protocol. Participants were asked at baseline to select four health‐related behaviors from a list of options. The behaviors fully crossed complexity (i.e., simple vs. complex) with establishment (i.e., nonestablished = performed <6 months; established = performed >6 months) to create simple nonestablished, simple established, complex nonestablished, and complex established conditions. Participants self‐declared their behaviors as established or not in response to a prompt that defined established behaviors as those that have been enacted for at least 6 months (based on the threshold for maintenance in the Transtheoretical Model of health; Prochaska & DiClemente, 1982). We do not assume that having established a behavior for 6 months is diagnostic of a habit. We asked participants to nominate established and nonestablished behaviors to ensure sufficient variance in habit strength across the selected behaviors, but habit strength itself was measured using validated instruments (see Self‐Reported Behavioral Automaticity Index in materials).

**FIGURE 2:**
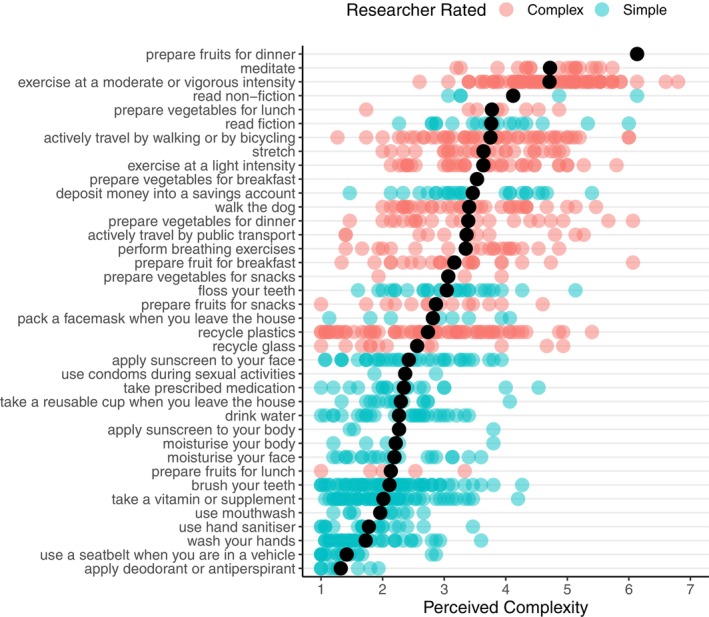
Participant self‐reported perceived complexity and researcher‐rated complexity for each behavior, ranked by the mean level of complexity across all datapoints shown by the black circles.

The list of both simple and complex behaviors was generated in line with recommendations from Phillips and Mullan (2022) insofar that the simple behaviors require fewer preparation steps and take less time to engage in than complex behaviors. Additionally, the complex behaviors all had meaningfully separatable actions, whereas the simple behaviors did not. Choices of simple behaviors included, for example, handwashing, flossing, and taking prescribed medication, while choices of complex behaviors included exercise at a moderate or vigorous intensity, active travel by walking or bicycling, prepare vegetables for lunch, and recycling plastics (see Figure 2 for a complete list of behaviors). Participants were also able to choose the option “other” and self‐specify a behavior that was not listed. Participants were told that this behavior should be a positive health behavior or one that enhances wellbeing. Participants were told that a simple behavior “is one that can be done without much planning and is not very challenging” and a complex behavior “is one that usually requires some planning and can be challenging.”

The order in which behavioral establishment for each type of behavior (i.e., complex or simple) was displayed was counterbalanced. Immediately after identifying each type of behavior (i.e., simple vs. complex and established vs. nonestablished) participants answered a battery of self‐report measures pertaining to said behavior, with the selected behavior inputted into the questionnaires where appropriate:

*Perceived Behavioral Complexity* was measured using five items from Boynton (2005; e.g., “In general, how challenging is [the behavior] for the average person?” and “How complex is [the behavior]”). Items were rated on a seven‐point Likert‐type scale with lower scores being indicative of lower perceived behavioral complexity.

*Instigation Habit Strength* was measured using the Self‐Reported Behavioral Automaticity Index (Gardner et al., 2012). The four items (e.g., “… I do automatically” and “… I do without thinking”) were preceded with the item stem “Deciding to [behavior] is something…” Items were rated on a five‐point Likert‐type scale with lower scores being indicative of lower levels of habit development, and an average score of four or more (i.e., a response option of agree) being indicative of habit development.

*Goal‐directed Strategies* were measured by having participants choose whether they engaged in any of 19 strategies over the previous 7 days pertaining to the specified chosen behavior (e.g., “I reduced or removed distractions or temptations,” and “I talked to myself to motivate me”) that were developed by Hennecke et al. (2019). One item was created for the purpose of the present study: “I did not use any of these strategies. I did it without thinking,” resulting in 20 options for participants. This option was created to allow participants to provide a meaningful response to advance the survey even in a case where they use no strategies, and this option was removed from the analyses (strategy count of zero if they only ticked this last option). All strategies were presented to participants for each behavior at each survey, and they were able to freely select as many that applied to their past week of engaging in that behavior. Other authors have used strategy checklists that are both shorter (e.g., Milyavskaya et al., 2021) and somewhat longer than our own (up to 25; cf., Bürgler et al., 2021; Bürgler & Hennecke, 2023).While no definitive, exhaustive list of self‐regulation strategies currently exists, our list provided good coverage of the stages of the process model of self‐regulation and was previously validated to study behavioral engagement (Hennecke et al., 2019).

*Behavioral Commitment* was assessed using two questions adapted from Milyavskaya et al. (2015). These questions were: “How committed do you feel toward your goal to perform this behavior” (1–7, Not at all committed – Extremely committed) and “Is your goal to perform this behavior something that you are working towards?” (1–7, Not at all—Very much).

*Behavioral Success* was assessed using two questions: (1) “Over the past 7 days I was satisfied that I completed this behavior to the appropriate degree,” and (2) “Over the past 7 days, I successfully enacted the behavior.” Both questions were answered on a seven‐point Likert scale ranging from “Strongly Disagree” to “Strongly Agree.”

For the two weekly surveys, instigation habit strength and goal‐directed strategy use were also measured for all four behavior types. Similar to baseline, the selected behavior was input into the questionnaires, where appropriate.

### 2.3 Statistical analysis

Data was analyzed using multilevel models with each datapoint nested within each participant. Random intercepts were included in every model for each participant. Random slopes were also used in models using the normal distribution. None of the models aggregated data across time points (minimum of 2855 datapoints, with three observations per participant per analysis cell). Contrast codes (−1 vs. 1) were used for all binary categorical predictors and continuous predictors were grand‐mean centered. Unstandardized betas are presented with standard errors, p‐values, and 95% Confidence Intervals [CIs] for each model. Significant interactions were described using predicted means and their 95% CIs (Cumming, 2014). Repeated‐measures *t*‐tests include Cohen's *d* in addition to its 95% CIs.

### 2.4 Open Science statement

Preregistration, code, and datasets for this study can be found on OSF (https://osf.io/p3mfy/; https://osf.io/5bu38). A priori analysis plans included *t*‐tests and multilevel models that tested equivalent hypotheses. Multilevel models are reported here, with the *t*‐tests available online (see Tables S2 and S3; https://osf.io/b7pvy). Absolute deviation from the median was preregistered to exclude outliers (Leys et al., 2013). However, this approach was not appropriate for the nonaggregated data as the mean absolute deviation was zero. As multilevel models are robust to extreme values, outlier exclusion was not applied to the below analyses. As the strategy variable was a count variable that included many zeros, a Poisson distribution that is more appropriate for this datatype was used (not preregistered). A preregistered model including habit strength as a continuous predictor and complexity as a categorical predictor also supported our hypotheses but was omitted from the manuscript for the sake of brevity. The following links can be used to access the open analysis code (https://osf.io/s9fcq) and dataset (https://osf.io/2y9cs). The Strengthening the Reporting of Observational Studies in Epidemiology (i.e., STROBE) checklist for observational studies was used at the data reporting stage to ensure transparent reporting (von Elm et al., 2014).

## 3 RESULTS

### 3.1 Measures and manipulation checks

Descriptive statistics and reliability for all measures are presented in Table S1 (https://osf.io/b7pvy). Scale measurements were reliable at each timepoint and for each of the four behavioral categories. Cronbach's alpha for the Automaticity Index ranged from 0.93 to 0.98, and from 0.77 to 0.90 for Perceived Behavioral Complexity. Test–retest reliability was assessed using intraclass correlations based on the average scores for each instrument's items at each wave of our study (i.e., one score per instrument per timepoint), ranging from 0.86 to 0.90 for the Automaticity Index, and between 0.87 and 0.88 for Perceived Behavioral Complexity.

Confirming the success of our manipulations, participants rated complex behaviors as being significantly more complex than simple behaviors, both when behaviors were established (complex: *M* = 3.56, *SE* = 0.08; simple: *M* = 2.24, *SE* = 0.06, *t*(236) = 14.81 *p* < 0.001, *d* = 0.96, 95% CIs [0.81, 1.12]) and nonestablished (complex: *M* = 3.69, *SE* = 0.08, simple: *M* = 2.38, *SE* = 0.06, *t*(236) = 15.24 *p* < 0.001, *d* = 0.99, 95% CIs [0.83, 1.15]). Figure 2 shows the participant self‐reported perceived complexity ratings for every behavior in our study, showing that researcher‐defined complex behaviors (in red) were largely associated with higher ratings of perceived complexity than behaviors that were defined by the experimenter as simple (in blue). That said, there were cases of disagreement between researcher‐defined complexity and the participants' perceived complexity, and considerable variance between participants in levels of perceived complexity. These factors highlight the importance of testing our hypotheses using both researcher‐defined complexity and participant‐rated perceived complexity.

Participants reported higher success executing simple established behaviors (*M* = 6.24, *SE* = 0.06) than simple nonestablished behaviors (*M* = 5.44, *SE* = 0.10), *t*(236) = −7.56, *p* < 0.001, *d* = −0.49, 95% CIs [−0.63, −0.36], and higher success for complex established behaviors (*M* = 5.38, *SE* = 0.10) than complex nonestablished behaviors (*M* = 4.77, *SE* = 0.10), *t*(236) = −4.78, *p* < 0.001, *d* = −0.31, 95% CIs [−0.44, −0.18]. Participants also reported higher success implementing simple established behaviors compared to complex established behaviors, *t*(236) = 8.39, *p* < 0.001, *d* = 0.55, 95% CIs [0.41, 0.68], and implementing simple nonestablished behaviors than complex nonestablished behaviors, *t*(236) = 6.18, *p* < 0.001, *d* = 0.40, 95% CIs [0.27, 0.53].

Participants ratings of commitment did not differ between established (*M* = 5.37, *SE* = 0.07) and nonestablished (*M* = 5.28, *SE* = 0.70) simple behaviors, *t*(236) = −1.46, *p* = 0.15, *d* = −0.01, 95% CIs [−0.22, 0.03]. Participants reported more commitment to established complex behaviors (*M* = 5.53, *SE* = 0.07) than to nonestablished complex behaviors (*M* = 5.32, *SE* = 0.08), *t*(236) = 2.50, *p* = 0.013, *d* = 0.16, 95% CIs [0.03, 0.29]. We controlled for these differences in commitment in an exploratory analysis, and this did not change the results of any models presented in the paper (see Table S4).

**FIGURE 3:**
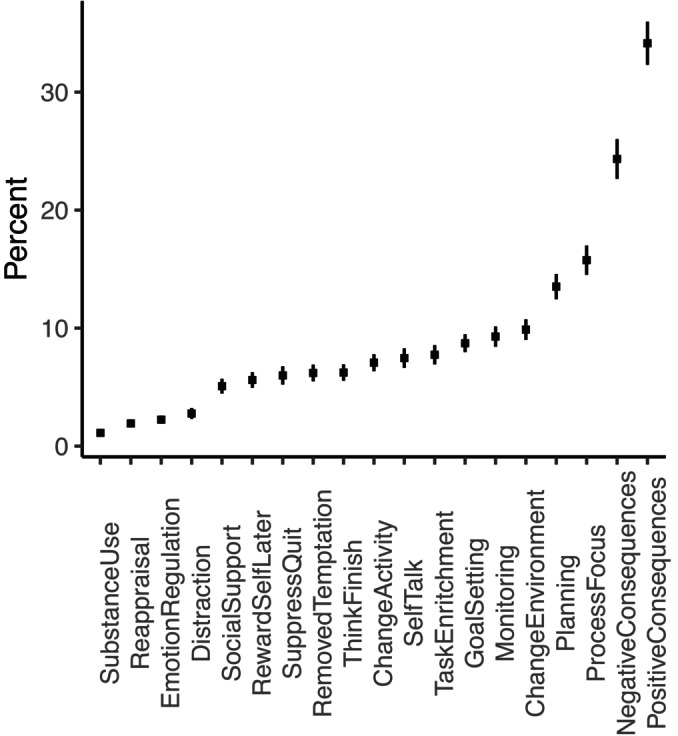
Strategy popularity in ascending order. Error bars depict standard errors.

It was also observed that participants used a wide range of strategies. Figure 3 shows the average extent to which each strategy was used on each occasion that participants were asked. As with previous work using these strategy options during experience sampling (Hennecke et al., 2019), the most popular strategy in our sample was “I reminded myself why I perform the activity and thought of its positive consequences” (reported on 34% of occasions). The next four most popular strategies were thinking of the negative consequences of not enacting the behavior (24%), adopting a process focus by deliberately focusing attention on task performance (16%), planning for a specific time to engage in the activity (14%), and changing the environment where the activity was performed (10%). The three least popular strategies were taking a substance or drug to change performance (1%), changing thoughts about the behavior (2%), and changing feelings (e.g., trying to stay in a good mood; 2%). Thus, our findings replicated the most popular strategies from two recent studies (i.e., Hennecke et al., 2019; Wenzel et al., 2024), although the exact rank order of less popular strategies differed between studies. These differences might arise because self‐regulation strategies choice is sensitive to the personal and situational factors that differ between studies (Hennecke & Bürgler, 2020), or might be related to differences in reporting method (weekly reports in our study vs. experience sampling measures in past studies), or in the instability of rank‐ordering when many strategies have similar prevalence (i.e., all but our top four).

### 3.2 Instigation habit strength (hypothesis 1)

We tested the effect of behavioral development (nonestablished: −1; established: 1), complexity (simple: −1; complex: 1), and their interaction. Instigation habits were lower for complex than for simple behaviors, *b*(237.00) = −0.36, *SE* = 0.03, *p* < 0.001, 95%CIs [−0.42, −0.30], and higher for established than nonestablished behaviors, *b*(237.00) = 0.29, *SE* = 0.04, *p* < 0.001, 95%CIs [0.22, 0.36]. Behavioral complexity also interacted with behavioral establishment, *b*(2140.19) = −0.10, *SE* = 0.01, *p <* 0.001, 95%CIs [−0.12, −0.07]. As predicted, established behaviors were associated with increased instigation habit strength for both simple behaviors (nonestablished: *M =* 2.75, *SE* = 0.07, 95% CIs [2.62, 2.89]; established: *M* = 3.53, SE = 0.07, 95% CIs [3.39, 3.67]) and for complex behaviors (nonestablished: *M =* 2.22, *SE* = 0.07, 95% CIs [2.08, 2.36]; established: *M* = 2.61, SE = 0.07, 95% CIs [2.48, 2.75]). Complex nonestablished behaviors had a lower instigation habit strength than all others, whereas simple established behaviors were associated with the highest instigation habit strength.

### 3.3 Strategy count (hypotheses 2 and 3)

We next tested hypothesis two, that complex behaviors would be associated with increased use of self‐regulation strategies compared with simple behaviors, hypothesis 3a that strategy use would be nonzero for even instigation habits related to simple behaviors; and hypothesis 3b that self‐regulation use would increase for habitually instigated behaviors of increasing complexity. In a preregistered model, we included behavioral establishment (nonestablished: −1; established: 1), complexity (simple: −1; complex: 1; see Model 1 in Table S5), and their interaction. Here, the interaction between complexity and establishment was statistically significant, *b* = 0.05, *SE* = 0.01, *Z* = 3.68, *p* < 0.001, 95% CIs [0.02, 0.08], in addition to the effects of complexity, *b* = 0.23, *SE* = 0.01, *Z* = 15.68, *p* < 0.001, 95% CIs [0.20, 0.26], and establishment, *b* = −0.11, *SE* = 0.01, *Z* = −7.93, *p* < 0.001, 95% CIs [−0.14, −0.09]. Estimated marginal means were calculated to probe the interaction between complexity and establishment. Fewer strategies were used for simple established (*M* = 0.83, 95% CIs [0.73, 0.95]) than simple nonestablished behaviors (*M* = 1.16, 95% CIs [1.03, 1.32]). In contrast, we did not observe a significant difference in strategy count between complex established behaviors (*M* = 1.46, 95% CIs [1.29, 1.65]) and complex nonestablished behaviors (*M* = 1.65, 95% CIs [1.46, 1.86]). These findings suggest that the extent to which established complex behaviors require support from self‐regulatory processes does not differ from the extent that these processes are engaged for similarly complex novel behaviors.

**FIGURE 4:**
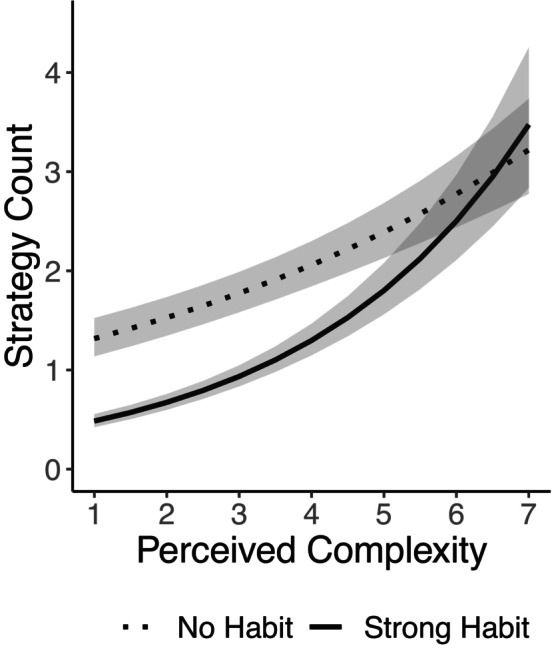
Poisson model with continuous predictors of rated complexity and habit strength. “No Habit” (dashed line) is plotted at rating of one out of five habit strength, and “Strong Habit” is plotted at four out of five habit strength. Shaded area depicts 95% CIs.

This initial categorical model does not completely test our hypotheses because established behaviors did not always reach automaticity levels typically diagnostic of an instigation habit (i.e., ≥ 4 on the Automaticity Index). A further fully continuous model tested the relationships among participant‐rated instigation habit strength, participant‐rated perceived complexity, and strategy use. Here, both automaticity and perceived complexity were operationalized as continuous self‐reported variables (see Table S5, Model 2). This model allows for the estimation of strategy use in cases where instigation habits are particularly strong for behaviors of increasing levels of perceived complexity. Results indicated a significant interaction between instigation habit strength and perceived complexity, *b* = 0.06, *SE* = 0.01, *Z* = 6.67, *p* < 0.001, 95% CIs [0.04, 0.08]. The effects of perceived complexity, *b* = 0.26, *SE* = 0.01, *Z* = 18.91, *p* < 0.001, 95% CI [0.23, 0.28], and rated instigation habit strength, *b* = −0.21, *SE* = 0.02, *Z* = −13.77, *p* < 0.001, 95% CIs [−0.24, −0.18] were also significant. To explore the interaction between self‐rated complexity and instigation habit strength, this model was predicted across all levels of perceived complexity for nonhabits (average of one out of five on the Automaticity Index) and strong habits (average of four out of five on the Automaticity Index) (see Figure 4 and Table S6).

As predicted, strategy use was higher for nonhabitually instigated behaviors (*M* = 1.32, 95% CIs [1.14, 1.52]) than for habitually instigated behaviors (*M* = 0.48, 95% CIs [0.42, 0.55]) when perceived complexity was low (i.e., one on the Perceived Complexity Scale). Confirmation of hypothesis 3b, that self‐regulatory support would become increasingly frequent in the context of instigation habits for complex behaviors relative to simple behaviors, is supported by inspecting the slope of strategy use for behaviors associated with strong habits. Within the context of habitually instigated behaviors scores at the midpoint of the Perceived Behavioral Complexity scale (i.e., four) were associated with elevated strategy use (*M* = 1.30, 95% CIs [1.15, 1.47]) compared to low complexity habitually instigated behaviors (*M* = 0.48, 95% CIs [0.42, 0.55]). In fact, each consecutive one‐point increase from scores of one to five out of seven on the Perceived Behavioral Complexity scale was associated with a significant increase in reported self‐regulation strategy use (see nonoverlapping 95% CIs, see Table S6), with no significant difference observed between six and seven where strategy use was particularly high (e.g., at six out of seven, *M* = 2.50, 95% CIs [2.11, 2.97]). Thus, while strategy use was particularly high at the top of the scale, strategy use was already significantly elevated for habitually instigated behaviors of average (*M* = 2.97 in our dataset) compared to low complexity.

Further support for hypothesis 3 was found by exploring the difference in strategy use between habitually instigated and nonhabitually instigated behaviors across complexity levels. While this difference between means was present up‐to‐and‐including a perceived complexity rating of five out of seven, no difference in mean strategy use between habitually instigated and nonhabitually instigated behaviors was present when complexity ratings were higher (see Figure 4). These findings suggest that self‐regulation strategies are not used significantly less for highly habitual instigation of complex behaviors than they are for nonhabitual instigation of complex behaviors when these actions are rated at the top of the self‐rated complexity scale. Some caution should be taken when interpreting these findings, however, as there were fewer datapoints at the very top of the complexity scale that were also rated as highly habitual. It should be noted that confirming hypothesis 3 does not depend on identifying no significant difference in strategy use between instigation habits and nonhabits at the top of the complexity scale.

## 4 DISCUSSION

Promoting regular engagement in complex health behaviors such as exercising, healthy eating, active traveling, and recycling is of interest across areas of Psychology, particularly because encouraging these behaviors has wide potential impact in terms of reducing healthcare costs (Scarborough et al., 2011) and tackling climate change (Brand et al., 2021). The current study supported a novel hypothesis – that engagement in complex healthy behaviors recruits support from a suite of self‐regulatory processes, even when those behaviors are instigated in a strongly habitual manner. Primary support for this hypothesis comes from the finding that strong habits (i.e., ≥ 4 on the Self‐Reported Behavioral Automaticity Index) that are associated with even moderately elevated levels of behavioral complexity were correlated with increased use of self‐regulation strategies compared to equally strong instigation habits for simpler behaviors. Further support for this hypothesis came from the finding that there were no differences in strategy use between strongly habitual and nonhabitual behaviors for the most complex actions.

### 4.1 Theoretical implications

Our findings shed new light on the interplay between habit‐mediated and goal‐directed processes. Existing accounts suggest self‐regulation should be inversely correlated with habitual processes because strongly automated behaviors are theorized to operate without the need for goal‐directed processes (Zhang et al., 2022). Many theoretical accounts suggest that goal‐directed and habit‐mediated processes are separable determinants of behavior that interact only in limited circumstances (Gardner et al., 2020; Wood et al., 2022). However, our results align with the theoretical proposition made by Hagger (2020) and Phillips and Mullan (2022) by providing empirical support for the theoretical claim that interactions between habitual and goal‐directed processes are more commonplace in the context of increasingly complex behaviors. These results point to a qualitative distinction where relatively simple behaviors (e.g., taking a daily supplement) can be instigated relatively automatically in the way that habits are typically characterized, whereas support from self‐regulation strategies becomes increasingly frequent for increasingly complex behaviors (e.g., exercise, preparing vegetables for dinner), even when the habit to instigate the behaviors is equally strong.

Complexity can be defined a priori based on objective features of the behavior, such as the number of separable steps to execute the behavior, or the behavior's temporal duration (Phillips & Mullan, 2022), or, alternatively, as a person's subjective perception of multiple dimensions, such as self‐reported challenge, attentional demands, complexity, necessary planning, or duration (Boynton, 2005; Rebar et al., 2023). Inspection of Figure 2 indicates that participants did not always agree with a priori researcher‐defined complexity, and participant‐rated complexity also varied greatly within each type of behavior. “Preparing fruits for lunch,” for example, was defined by the researchers as a complex behavior, yet the participants rated this as a relatively simple task. This points to the limits of our ability to define any specific behavior as unequivocally “complex” or “simple.” Indeed, “preparing fruits for lunch” could be as simple as grabbing an apple, or more complex if the person rinses, peels, deseeds, and chops the fruit. In contrast, some behaviors did seem more straightforwardly complex (e.g., vigorous exercise) or simple (e.g., wearing a seatbelt). Although some differences existed between participants' perceived complexity and researcher‐defined complexity, our findings were robust and consistent across objective and subjective definitions. Thus, our findings provide robust support that habitual instigation and goal‐directed self‐regulation act together to produce complex behavioral engagement.

When considering self‐regulation in the context of instigation habits, it is tempting to view increased strategy use as a direct reflection of the multiple steps involved in executing complex behaviors. However, self‐regulation strategies do not map onto behavioral steps directly, but instead reflect more general goal‐related processes (e.g., reminding yourself why you do it) that can indirectly encourage the instigation or execution of goal consistent behaviors. Such indirect goal‐directed processing might be unnecessary for simple behaviors associated with instigation habits because these habits potentially have a one‐to‐one mapping with action (i.e., an instigation cue leads to taking a pill). Conversely, more self‐regulation may be required in the context of complex behaviors, even if they are habitually instigated. Furthermore, complex behaviors, even when established for at least 6 months, were typically associated with weaker instigation habit strength than simpler behaviors. As such, one might reason that complex behaviors require more self‐regulatory support simply because they are not as habitual. Our findings provide direct evidence against such an assertion; increasing levels of complexity (both objective and subjective) were associated with increasing strategy use even for behaviors that participants rated as being instigated with high levels of automaticity using established cut‐offs on the validated Automaticity Index (e.g., Phillips et al., 2019). This increased strategy use was particularly pronounced at high levels of complexity, but was also present at moderate perceived complexity levels. Thus, even behaviors of modest complexity are supported by increased strategy use compared to behaviors of low complexity.

The Self‐Reported Automaticity Index and similar measures are widely used to diagnose habits across many behavioral domains (Hagger et al., 2023). Our results suggest that identifying a behavior as a habit on this scale does not necessarily mean that the behavior is engaged in an automatic reflex‐like manner. Instead, our results suggest that habitually instigated behaviors with even relatively average levels of complexity will be routinely supported by self‐regulation strategies, with this extending to the use of multiple self‐regulation strategies (identified as polyregulation, Ford et al., 2019) when behaviors are particularly complex.

Although our results point to collaboration between self‐regulation and habitual processes for complex behaviors, the implementation of self‐regulation strategies was lower for habitually instigated than nonhabitually instigated behaviors when complexity was moderate. As such, our findings do not contradict previous suggestions that habit formation reduces the prevalence of goal‐directed processes in the execution of behavior (Overmeyer et al., 2020; Triandis, 1977; Zhang et al., 2022). Our results add to this previous literature by suggesting that the extent that the formation of an instigation habit is associated with a reduction in self‐regulation for a specific behavioral domain is moderated by the complexity of that behavior.

Our results highlight the practicality of considering behavioral complexity when designing health‐behavior change interventions. Behavior change techniques targeting habit formation, such as action planning, may sufficiently support the change and maintenance of simple health behaviors, like flossing one's teeth (Michie et al., 2013). However, a toolbox that targets self‐regulation strategies, such as seeking social support, monitoring progress, and self‐talk, in addition to habit formation, may be more effective to support the instigation and maintenance of complex behaviors (Fujita et al., 2020). Future intervention development could adopt a hybrid approach that acknowledges the complementarity of instigation habits and self‐regulation strategies to help individuals integrate complex behaviors into their lifestyle. The strategies included in our study were relatively wide‐ranging and covered all stages in the process model (Duckworth et al., 2016). Nevertheless, other recent studies have used longer lists of strategies (Bürgler et al., 2021; Bürgler & Hennecke, 2023). Future research should refine taxonomies of self‐regulation strategies to provide as complete a list as possible. One challenge might come from communicating ever‐larger lists of self‐regulation strategies during an intervention.

### 4.2 Limitations and future directions

Our investigation was intended to be generative, aiming to stimulate novel lines of enquiry at the intersection of instigation habits and self‐regulation. Future research should examine the importance of habitual and self‐regulatory processes with regards to behavioral complexity using a randomized experimental paradigm. This research would best be served by taking a Multiphase Optimization Strategy approach (Collins et al., 2007), using a full factorial experimental design whereby each intervention component is present or absent, to understand the effectiveness of *each* intervention component for the changing and maintenance of both simple and complex behaviors. Such future research could extend the current paradigm to include objective measures of behavioral engagement, such as measures of physical activity through accelerometers. Considering our results, we anticipate that individuals who were assigned to use self‐regulatory strategies alongside a habitual intervention to encourage complex health behaviors would be more successful at doing so than individuals who use no strategies to support engagement.

Self‐regulation strategies could themselves become highly routinized to an extent that a person might learn to habitually use specific self‐regulation strategies in the context of specific complex behaviors (so‐called “effortless” self‐regulation, Gillebaart & de Ridder, 2015). In our study, the three most popular strategies involved two strategies about thinking of the future positive or negative consequences of the behavior (e.g., “I reminded myself why I perform the activity and thought of its positive consequences”) and a third in which participants adopted a process focus (“I focused my attention on the activity itself and the way I was performing it”). It remains possible that people learn to engage in these self‐regulation processes relatively habitually in the context of complex behaviors, and future research could investigate this by exploring the reported automaticity of self‐regulation strategy use.

A limitation of the presented study is the general nature of the self‐regulation questionnaire which involves both items pertaining to the instigation (e.g., “I made a plan or set a specific time for engaging in the activity”) and execution (e.g., “I added something positive to the activity to make it more pleasant”) of behavior. Thus, future research should explore when and where in the behavioral sequence do self‐regulation strategies support the enactment of habitually instigated behaviors. It might be expected, for example, that goal‐directed processes encourage behavioral instigation at times when habit cues are disrupted, when motivation is particularly low, or when unexpected events conflict with the instigation of a behavior (Charlesworth et al., 2022; Gardner, 2015; Triandis, 1977). Barriers could equally interfere with the actual execution of a behavior after habitual instigation, providing another avenue through which goal‐directed processes might keep behaviors on track. Ongoing research could explore these possibilities using more intensive longitudinal methods (e.g., ecological momentary assessment) to understand when and where self‐regulation strategies support habitually instigated behaviors. We would predict that different strategies might be used in different contexts depending on the nature of the barrier to behavioral instigation (e.g., using reappraisal to encourage a run despite bad weather) or execution (e.g., using task enrichment with music to maintain a run that was habitually instigated).

Finally, our participants reported behaviors over one‐week periods. This method, we suggest, was appropriate because some of the behaviors in our survey (e.g., using public transport, reading a novel) might not happen with a frequency that necessitates ecological momentary assessment (EMA). We also note that the relative popularity of strategies (particularly at the top of the scale) in our study overlaps considerably with analogous EMA findings (Hennecke et al., 2019; Wenzel et al., 2024). That said, the retrospective nature of our design might result in the reporting the most memorable episodes of self‐regulation. While we do not think that this would hinder our conclusions, particularly because we suspect this would lead to an underreporting of self‐regulation in the context of complex behaviors, future work could use EMA to detect more subtle and fluctuating interactions between habits and self‐regulation. From the current data, for example, it is not currently possible to know if self‐regulation is used in every instance of habitually instigated complex behavior, or, instead, if self‐regulation is only used as needed. EMA approaches could be used to further explore these dynamics.

## 5 CONCLUSION

In conclusion, we tested and supported a novel hypothesis that for complex behaviors, engagement likely needs to be supported by self‐regulatory strategies, even when these behaviors are well‐established and habitually instigated. Although instigation habits have been shown to be critical for the maintenance of health promoting behaviors (e.g., Phillips & Gardner, 2016), habitually deciding to engage in or habitually starting to engage in a complex behavior is likely not enough to ensure that the behavior is executed to completion. Despite aspects of a complex behavior having the ability to be habitual (Gardner, 2016), our study indicates that self‐regulation strategies may be a fruitful means of ensuring full behavioral execution even in the presence of strong instigation habits.

## AUTHOR CONTRIBUTIONS

Blair Saunders: Conceptualization; Methodology; Formal Analysis; Data Curation; Writing; Visualization; Project Administration; Funding Acquisition. Kimberly More: Conceptualization; Methodology; Formal Analysis; Data Curation; Writing; Project Administration; Funding Acquisition.

## FUNDING INFORMATION

This work was supported through the University of Dundee Strategic Development and Impact Funding pathway.

## CONFLICT OF INTEREST STATEMENT

None of the authors have a conflict of interest to disclose.

## ETHICS STATEMENT

This study was approved by the School of Humanities, Social Sciences, and Law ethics committee at the University of Dundee.

## Data Availability

The following links can be used to access the open analysis code (https://osf.io/s9fcq) and dataset (https://osf.io/2y9cs).

## Endnote

^1^
The hypotheses and analysis strategy were preregisted prior to data collection (https://osf.io/5bu38/). This preregistration follows the criteria for a Preregistered + Analysis Plan badge from the Centre for Open Science.
